# Study of Electrolyte Changes in Patients with Prolonged Labour in Ikot Ekpene, a Rural Community in Niger Delta Region of Nigeria

**DOI:** 10.5402/2012/430265

**Published:** 2012-12-17

**Authors:** E. I. Ekanem, A. Umoiyoho, A. Inyang-Otu

**Affiliations:** ^1^Department of Obstetrics and Gynaecology, University of Calabar Teaching Hospital, Cross River State, Calabar, Nigeria; ^2^Department of Obstetrics and Gynaecology, University of Uyo Teaching Hospital, Akwa Ibom State, Uyo, Nigeria; ^3^Department of laboratory Sciences, General Hospital Ikot Ekpene, Akwa Ibom State, Ikot Ekpene, Nigeria

## Abstract

*Background*. Prolonged obstructed labour is a major cause of maternal and perinatal morbidity and mortality especially in the developing countries of the world, where the incidence is high. These complications are partly attributed to the metabolic and electrolyte derangements that are often associated with this problem. It is, therefore, important to evaluate the metabolic and electrolyte changes of these patients in a rural community in a developing country. *Objective*. To compare the electrolyte changes, maternal, and perinatal outcomes in patients with prolonged obstructed labour with that of normal labour in General Hospital Ikot Ekpene, Akwa Ibom State, Nigeria. *Patients and Methods*. This is a prospective cross-sectional case control study conducted in the Labour Ward of the General Hospital Ikot Ekpene to compare the electrolyte levels and perinatal outcome of 95 pregnant women who had prolonged labour with 105 women who had normal labour within the same period. *Main Outcome Measures*. Electrolyte changes, ketonuria, maternal complications, and perinatal outcome. *Results*. The majority of women with prolonged labour (91.6%) had major surgical interventions requiring anaesthesia. Perinatal death occurred in 12.6%, and a major life-threatening maternal complications (including two deaths) occurred in 13.7% of those with prolonged labour compared to 2.9% (with no death) in those with normal labour. Significant abnormal electrolyte changes included hyperkalemia, high urea, and creatinine as well as low bicarbonate levels were recorded. Metabolic abnormality was shown by ketonuria in 91.1% of the patients compared to 1.9% in women with normal labour. *Conclusion*. Women with prolonged labour in Ikot Ekpene have significant electrolyte and metabolic changes which impact adversely on the maternal and perinatal outcomes of the pregnancy. Effort should be made to correct these electrolyte and metabolic abnormalities during resuscitation of the woman in order to reduce the complications associated with such derangements.

## 1. Introduction

Obstructed labour ranked 41st in Global Burden of Disease (GBD) in 1990, representing 0.5% of the burden of all conditions and 22% of all maternal conditions [1]. It is well documented that prolonged obstructed labour is still a major cause of unacceptable high maternal and perinatal morbidity and mortality globally especially in the developing countries of the world. In most Sub-Saharan Africa countries [2, 3], it has become a major public health problem, where huge scarce public resources are being used to manage these highly preventable obstetric problem and its associated complications [4].

A number of reasons have been advanced for the high prevalence of this problem in Nigeria including ignorance, poverty, poor public transport system, and inefficient health institutional facilities and management [4, 5]. Therefore, most of women are usually unbooked, primigravida from poor socioeconomic and educational background as well as those whose labours were conducted by untrained personnel [6, 7]. Also, the incident of this problem is likely to be high in the rural communities, where these contributing factors are not only more but influenced by adverse cultural/religious believes [6, 8, 9].

Most of the deaths from obstructed labour are largely associated with hemorrhage, ruptured uterus, infections, metabolic, and electrolyte derangements [10–12]. The fluid and electrolyte changes in obstructed labour are well documented and may be due to abnormal metabolic activities, prolonged starving of the patient from food and water, excessive uterine contractions and muscular activities, infections with associated high temperature and subsequent loss of fluids, and maternal exhaustion [13–16]. Generally, electrolyte derangements may be compounded by inefficient fluid management in labour in the hospitals [14, 15]. But, in the rural community, the majority of labour and deliveries are attended to by untrained personnel outside the hospital [6, 9, 12]. In these area, however, the fluid and electrolyte problem is largely affected by large intake of herbal/other medicinal preparations of unknown efficacy and safety given by the traditional birth attendants. Also, prolonged starving may be exaggerated by fasting and prayer sessions in labour prescribed by the religious leaders for spiritual guidance since the prolonged labour is often linked to sins committed by the woman during pregnancy [9, 10]. 

Electrolyte derangement is, therefore, a common complication of prolonged obstructed labour in the rural community in the developing country [5, 10, 14]. These abnormalities can cause cardiac arrhythmias or cardiopulmonary arrest. Life-threatening arrhythmias are associated commonly with potassium disorders particularly hyperkalaemia, and less commonly with disorder of serum calcium and magnesium [17]. Among the electrolyte derangements, hyperkalaemia is the most common disorder associated with cardiopulmonary arrest. It is usually caused by increased potassium release from cells, impaired excretion by the kidney, or poor fluid management in labour [13, 14, 17]. This hperkalaemia is often easily corrected, and complications averted by administration of appropriate intravenous fluids [15]. 

In most cases, therapy for life-threatening electrolyte disorders is very urgent and may need to be started before laboratory results are available. Besides, in most rural communities in developing countries like Ikot Ekpene, laboratory facilities may not be readily available to assess the fluid and electrolytes derangement in women with obstructed labour even though there is compelling need to correct them to avoid the complications associated with the problem. 

Many studies have been conducted in our environment [6, 7, 9, 12] on obstructed labour but none have been done in the rural area in Akwa Ibom State to assess the metabolic and electrolytes changes in this group of women hence the need for this study. 

This study was therefore, carried out in the maternity section of the General Hospital Ikot Ekpene, a secondary health care facility in rural community in Akwa Ibom of Nigeria with the aims of assessing the socio demographic factors, maternal and perinatal outcome, and electrolyte changes in women with prolonged labour.

It is hoped that the outcome of this study will highlight the maternal and perinatal outcome as well as electrolyte changes in prolonged labour in this community. These will therefore, help in targeting interventions to improve the outcome. It would also help to create baseline values of the electrolyte changes in prolonged labour in this environment, where empirical treatment can be based when the laboratory results are not available for any reasons. 

## 2. Patients and Method

This is a case control cross-sectional observational study carried out in the Labour Ward of General Hospital Ikot Ekpene, Akwa Ibom State, over a three-year period of 1st January 2008 and 31st December, 2011.

The General Hospital is a secondary health care facility which was upgraded in 2008 to provide specialist health care services in the state. It is the only referral health care institution in the area for the public and private hospitals as well as any patient who present directly for care. It has an annual delivery of 2,400 and a caesarean rate of 30.1%.

Ikot Ekpene is one of the local government areas in the state with a population of about 500,000 people. The women are mainly subsistent farmers, petty traders, housewives, and civil servants with fertility rate of 7.1 and low female literacy level [18].

### 2.1. Ethical Approval

Written approval was obtained from the ethical committee of the institution and unwritten informed consent from the women after careful and personal discussions with each of them by the authors. 

### 2.2. Patient Recruitment

Women with a diagnosis of prolonged labour were included as cases. Pronged labour was defined as labour lasting more than 12 hours from the active phase irrespective of parity or causes.

### 2.3. Matching Criteria

Those with normal labour of equivalent maternal age, parity, gestational age, and social class who delivered within one week of the index case were selected as control. 

### 2.4. Exclusion Criteria

Women with preexisting metabolic and medical conditions in pregnancy, those on antibiotics therapy, intravenous infusions or other medications; HIV seropositive, women and those that the diagnosis was uncertain. Those who refused to give consent were also excluded but were assured and given appropriate management irrespective of the decision. 

### 2.5. Methods

Relevant medical history and physical examinations were carried out on all consenting women. Appropriate laboratory investigation and planned management were carried out and instituted according to the protocols in the hospital. For the purpose of the study, each woman was coded with unique number and separated as case and control which was communicated to all involved in the study. The relevant sociodemographic and reproductive information were obtained from the women on admission or within one week of delivery using pretested proforma prepared for the study. Other information was obtained from the operating Theatre Register, Labour Ward, Postnatal Ward, and Neonatal Unit records. 

#### 2.5.1. Collection of Specimen

After careful counseling, the women were allowed to lie on the lateral position in bed, and appropriate aseptic preparation of the right cubital fossa was carried out. Ten milliliters of venous blood was taken with a sterile hypodermic needle and syringes before any medication or intravenous infusion was started. This was put in labeled blood specimen container precoded for each woman. This was sent to the laboratory immediately for processing. 

After passing a Foley catheter, urine sample was taken and put in labeled urine container for each woman.

#### 2.5.2. Timing of Specimen Collection

The blood and urine samples were obtained from the patients on admission or within one hour of contact in the hospital.

#### 2.5.3. Processing of Specimen

The specimens were processed in the laboratory within one hour of collection by the same group of staff supervised by one of the authors to avoid inter observer errors. The electrolytes were analyzed using automated machine Roche Hitachi 917 Automated Chemistry Analyzer. Dip stick urine analysis to check for ketones was done using combi-9. 

A single baby weighing scale calibrated in kilograms was used to measure the weight of all the babies within 30 minutes of birth. APGAR's score was taken at one, five, and ten minutes of birth, respectively.

#### 2.5.4. Data Analysis

The data was analyzed using statistical software (SPSS version 18, Illinois) and was displayed in tables and figures. Significance level was considered at *P*  value < 0.05 at confident interval of 95%.

The results of this study form the basis of discussion, conclusion, and the recommendations made.

## 3. Definitions

 For the purpose of this study the following definitions were applicable

 Pronged labour was defined as labour lasting more than 12 hours from the active phase irrespective of parity or causes.
 Normal labour is defined as labour that stated spontaneously at term and lasted less than 12 hours in the active.
 Unbooked referred to women admitted in labour without antenatal care in recognized orthodox medical institution. It also included patients with antenatal but attempted to deliver with unskilled medical attendants and is only referred to the hospital at advanced stage of labour.
 Booked women are those who booked and deliver in the hospital and included those referred from other recognized medical institutions.

## 4. Results

A total of 8,640 deliveries were conducted during the period, and 115 were prolonged labour giving an incidence of 1 in 75 deliveries or 1.3%. However, 95 patients were eligible and included in the study for analysis.

Table 1 shows the sociodemographic features of patients with prolonged labour and controls. Though there was no significant differences (*P* = 0.2) in the maternal age of the patients compared to the controls, labor tended to be prolonged among the young (46.3% versus 11.4%) and older patients. Also, the incidence of prolonged labour was more in women with low formal educational background (76.8% versus 25.7% of those educated up to primary school). There was, however, no significant differences (*P* = 0.812) in educational attainment.

As shown in Table 2, there was no statistical significant difference in their parity (*P* = 0.585), gestational age (*P* = 0.45), and booking status (*P* = 0.275), but most cases of prolonged labour occurred in those with higher gestational age (39.7% versus 38.2%) and the unbooked patients (84.3% versus 23.9%).

Table 3 shows significant incidence of operative delivery (*P* ≤ 0.001) and major maternal complications (*P* ≤ 0.001) among those with prolonged labour. Most of the cases of prolonged labour had caesarean section (91.6% versus 13.3%) and serious maternal complications including 2 deaths (13.7% versus 2.9%) compared to those with normal delivery.

As shown in Table 4, there was significant longer duration of labour (*P* ≤ 0.001) among cases of prolonged labour (45.1 versus 8.3 hours) compared to those with normal labour. Also more babies had birth asphyxia and perinatal mortality as well as being larger at birth (*P* ≤ 0.001) than women with normal deliveries. 

In Table 5, there are significant changes in the electrolyte levels in the two groups. Women with prolonged labour had hyperkalaemia (mean 6.4 versus 4.1), high urea (mean 11.1 versus 4.3), and creatinine (mean 111.2 versus 62) as well as low bicarbonate levels (mean 17.6 versus 23.8). Also, there was significant number of women with prolonged labour who had ketonuria compared to those with normal labour (91.6% versus 1.9%). 

## 5. Discussion

The incidence of obstructed labour in General Hospital Ikot Ekpene in this study appears high when compared to findings in some area in Southern Nigeria but compatible with studies in Northern Nigeria [18, 19] and other developing countries [6, 10]. Generally, there is no clear definition of prolonged labour, and confusion of terms used by different authors remain [1, 2, 6, 8]. The finding in this study may be explained by the fact that it is a referral institution where difficult cases are pulled from different part of the area for specialized care. Besides, it may not even be a reflection of what is obtained in the general population as many may be dying without reaching the hospital. But, this actually emphasizes that the prolonged obstructed labour has become a public health problem despite the efforts put in place to attain the millennium development goals 4 and 5 [7, 19, 20].

As documented by others [1, 2, 6, 7], large proportions of our cases with prolonged labour were young, poorly educated, and unbooked women. But, a quarter of them in our study had good level of education. This latter group of women may be influenced by factors like adverse cultural and religious considerations to attempt delivery with unskilled personnel only to be referred when complications occur [9, 12, 20]. During the antenatal periods, the harmful effect of adverse practices and encouragement of good ones should form major part of the discussion as many of the patients in this study actually had antenatal care but attempt delivery outside the hospital. 

As in other studies [5, 6], abdominal operative delivery was the main mode of relieving the obstruction in Ikot Ekpene. In contrast to other findings in most sub-Saharan African countries; [10, 19, 20], however, 8.4% of our patients had vaginal deliveries, and major maternal complications were found only in 23.1%. This highlights the fact that some causes of prolonged labour may not necessarily lead to obstruction that require surgery and that careful assessment of the patients should always be undertaken before embarking on surgical delivery especially in our society, where subsequent delivery may be in the hospital [9, 12]. Also our center being a newly upgraded referral institution in the area was well equipped to manage these cases. 

The electrolyte changes in prolonged labour in this study included hypernatraemia, hperkalemia, and acidosis. The changes were directly related to the duration of labour. Some of these patients labour for many days in home without food or water and, therefore, presented in shock and severe dehydration. This latter condition could also reflect in the high level of ketonuria in most of our women with prolonged labour. These changes may also have effect on the perinatal outcome of the babies as shown by high incidence of birth asphyxia and other morbidities. Similar findings are also obtained by the studies in Nigeria and developing countries [14, 20]. This is however, different from hyponatremia and minimal changes in the metabolic and electrolyte status in labour commonly in developed world. Hyponatremia is often associated with liberal infusion of isotonic fluids or induction of labour with oxytocin or its toxicity [13]. Fluid and electrolyte changes in pregnancy, normal, and abnormal labour including puerperium are well documented [13–16]. Though this should guide the fluid and electrolyte management in these patients, they are, however, affected by many factors in our area which should be considered while appropriate therapies are administered. Excessive intake of herbal preparations and fasting from food and water for many days as therapy for prolonged labour are often given by the unskilled attendants in labour. Electrolyte abnormalities can cause cardiac arrhythmias or cardiopulmonary arrest. Life-threatening arrhythmias are associated most commonly with potassium disorders particularly with hyperkalaemia, and less commonly with disorders of serum calcium and magnesium. Among the electrolyte derangements, hyperkalaemia is the most common disorder associated with cardiopulmonary arrest. This should be considered and corrected before anesthetic agents are administered [17].

## 6. Conclusions

Obstructed labour is clearly associated with electrolyte derangements and poor fetomaternal outcomes compared to normal labour in this study. Effort should always be made to correct these electrolyte and metabolic abnormalities during resuscitation of the woman in order to reduce the complications associated with such derangements. The general public and the care givers should be educated on the dangers of prolonged labour and ways to prevent it. The training and effective utilization of composite partograph in labour in all tiers of health care levels in Nigeria should be encouraged, as this has been shown to significantly reduce incidence of prolonged labour. 

## Figures and Tables

**Table 1:** Sociodemographic characteristics of cases of prolonged labour and controls.

Variable	Cases *N* (%)	Control *N* (%)	All *N* (%)	*χ* ^2^	*P* value
	95 (100)	105 (100)	200 (100)		
Age (years)					
13–19	44 (46.3)	12 (11.4)	56 (28.0)	11.781	0.20
20–30	30 (31.6)	62 (59.0)	92 (46.0)		
31–40	17 (17.9)	24 (22.9)	41 (20.5)		
41–50	4 (4.2)	5 (4.7)	5 (2.5)		
Educational status					
None/primary	73 (78.0)	27 (25.7)	102 (51.0)	0.056	0.812
Above primary	11 (22.0)	78 (74.3)	97 (49.0)		

**Table:** (a)

Variable	Cases Mean (SD)	Control Mean (SD)	All Mean (SD)	*P* value
Age	17.4 (6.0)	18.7 (3.4)	18.1 (5.0)	0.183
Parity	0.8 (1.4)	0.9 (1.1)	0.7 (1.3)	0.585
Gestational age (weeks)	39.7 (1.2)	38.2 (1.3)	39.1 (1.3)	0.42

**Table:** (b)

Variable	Cases *N* (%)	Control *N* (%)	All *N* (%)	*χ* ^2^/fishers exact	*P* value
	95 (100)	105 (100)	200 (100)		
Booking status					
Booked	15 (15.7)	80 (76.1)	95 (47.5)	1.190	0.275
Unbooked	80 (84.3)	25 (23.9)	105 (52.5)		

**Table 3:** Mode of delivery and maternal outcomes of cases of obstructed labour.

Variable	Cases *N* (%)	Control *N* (%)	All *N* (%)	*χ* ^2^/fishers exact	*P* value
	95 (100)	105 (100)	200 (100)		
Mode of delivery					
Caesarean section	87 (91.6)	14 (13.3)	101 (50.5)		<0.001
Vaginal delivery	8 (8.4)	91 (87.7)	99 (49.5)		
Major maternal complications					
Complications	22 (23.1)	3 (2.9)	16 (8.0)		<0.012
No complications	73 (76.9)	103 (97.1)	184 (92.0)		

**Table 4:** Perinatal outcome of prolonged labour.

Variable	Cases Mean (SD)	Control Mean (SD)	All Mean (SD)	*P* value
Duration of labour (hour)	45.1 (19.3)	8.3 (2.0)	27.4 (22.5)	<0.001
APGAR at 1 minute	0.8 (1.0)	7.8 (1.0)	4.3 (4.0)	<0.001
APGAR at 5 minutes	0.9 (1.4)	9.7 (0.6)	5.6 (4.6)	<0.001
Birth weight (kg)	3.8 (0.4)	3.1 (0.3)	3.3 (0.7)	<0.001

Fetal outcome	*N* (%)	*N* (%)	*N* (%)	
Alive	83 (87.3)	104 (99.0)	187 (93.6)	<0.001
Perinatal death	12 (12.7)	1 (1.0)	13 (12.4)	

**Table 5:** Mean electrolyte levels between cases of prolonged labour and controls.

Variable	Cases Mean (SD)	Control Mean (SD)	All Mean (SD)	*P* value
K^+^ (mmol/L)	6.4 (0.2)	4.1 (0.3)	5.1 (1.0)	<0.001
Na^+^ (mmol/L)	150.6 (8.1)	131.7 (2.8)	142.2 (7.6)	<0.001
Cl^−^ (mmol/L)	111.9 (5.7)	99.0 (4.0)	103.4 (6.2)	<0.001
Urea (mmol/L)	11.1 (3.6)	4.7 (1.0)	6.9 (3.8)	<0.001
Bicarbonate (mmol/L)	17.6 (3.2)	23.8 (2.4)	21.2 (3.7)	<0.001
Creatinine (*μ*mol/L)	111.2 (12.7)	62.1 (12.2)	86.5 (25.4)	<0.001
Ketonuria				
Present	87 (91.6)	2 (1.9)	89 (44.5)	<0.001
Absent	8 (8.4)	103 (98.1)	111 (55.5)	
